# Study protocol: evaluation of the 0–5 public health investment in England – a mixed-methods study integrating analyses of national linked administrative data with in-depth case studies

**DOI:** 10.1136/bmjopen-2023-073313

**Published:** 2023-04-05

**Authors:** Katie Harron, Francesca L Cavallaro, Catherine Bunting, Amanda Clery, Sally Kendall, Rebecca Cassidy, Julie Atkins, Eirini-Christina Saloniki, Helen Bedford, Jenny Woodman

**Affiliations:** 1 UCL Great Ormond Street Institute of Child Health, University College London, London, UK; 2 The Health Foundation, London, UK; 3 Centre for Health Services Studies, University of Kent, Canterbury, UK; 4 Care City, London, UK; 5 Department of Applied Health Research, University College London, London, UK; 6 NIHR Applied Research Collaboration North Thames, London, UK; 7 Social Research Institute, UCL, London, UK

**Keywords:** Health policy, Health economics, Community child health, PUBLIC HEALTH

## Abstract

**Introduction:**

Health visiting is a long-established, nationally implemented programme that works with other services at a local level to improve the health and well-being of children and families. To maximise the impact and efficiency of the health visiting programme, policy-makers and commissioners need robust evidence on the costs and benefits of different levels and types of health visiting, for different families, in different local contexts.

**Methods and analysis:**

This mixed-methods study will analyse individual-level health visiting data for 2018/2019 and 2019/2020 linked with longitudinal data from children’s social care, hospitals and schools to estimate the association of number and type of health visiting contacts with a range of children and maternal outcomes. We will also use aggregate local authority data to estimate the association between local models of health visiting and area-level outcomes. Outcomes will include hospitalisations, breast feeding, vaccination, childhood obesity and maternal mental health. Where possible, outcomes will be valued in monetary terms, and we will compare total costs to total benefits of different health visiting service delivery models. Qualitative case studies and extensive stakeholder input will help explain the quantitative analyses and interpret the results in the context of local policy, practice and circumstance.

**Ethics and dissemination:**

The University College London Research Ethics Committee approved this study (ref 20561/002). Results will be submitted for publication in a peer-reviewed journal and findings will be shared and debated with national policy-makers, commissioners and managers of health visiting services, health visitors and parents.

**STRENGTHS AND LIMITATIONS OF THIS STUDY**

- This interdisciplinary study will integrate data from multiple sources including regression analyses, return on investment analysis and qualitative case studies to provide a rich and nuanced account of the impact and value of health visiting in England.
- Extensive stakeholder input and public involvement will enable us to focus the research on priority issues for policy-makers, practitioners and families, and to interpret the findings in the context of local area characteristics.
- Our analyses will use data on health visiting from the Community Services Dataset, restricted to areas for which sufficient data have been captured and linked to children’s health, education and social care data (Education and Child Health Insights from Linked Data).
- We will use propensity score matching to compare the outcomes of families with similar levels of underlying need who receive different levels of health visiting support, but there may be residual confounding due to unmeasured factors such as income or parenting style.

## Introduction

Health visiting is a long-established, national intervention that works with other services including general practitioners (GPs), paediatricians and social care to maximise the health and well-being of children. Health visitors lead the Healthy Child Programme (HCP) for children aged 0–5 years in England, providing a universal, preventive service to families that includes health screening, immunisation, health and development reviews and parenting advice and support.^1^ The health visiting model is designed to be ‘universal in reach—personalised in response’.^1^ It is underpinned by holistic needs assessment and relationship work with families, with health visitors offering guidance and interventions that are tailored to meet the needs of individual children and families.^1 2^

There are five universal mandated health visiting contacts: at 28 weeks pregnancy, 10–14 days after birth, 6–8 weeks, 9–12 months and 2–2.5 years. It is expected that most families will have most of their needs met by assessment, guidance and signposting within the five mandated contacts. Other families need more targeted support, including additional health visiting contacts and/or referral for interventions from other professionals such as early years, education, voluntary organisations, social services and GPs and primary care teams.^1^ Health visiting contacts can be face to face (at home or in a clinic or community centre), online, by telephone or by letter. Each contact may be delivered by the same or a different health visitor, or another member of the health visiting team such as a community staff nurse.

Health visiting service delivery varies across England, but little is known about the number or type of health visiting contacts that different families receive.^3 4^ Public Health England statistics suggest that not all children receive their mandated reviews: coverage is lower for ethnic minorities and children living in deprived areas.^5 6^ At the same time, two analyses using the Community Services Dataset (CSDS), the only national data on health visiting,^5–7^ suggest that children living in deprived areas receive more additional contacts.^7 8^ These nuanced findings highlight the need for careful analysis of administrative data to describe routine health visiting, distinguishing between mandated and additional contacts and applying robust methods for handling missing data.

There is a lack of evidence on the impact of health visiting on child outcomes in England. Within the HCP, the six high-impact areas for health visiting services are: supporting the transition to parenthood, supporting maternal and family mental health; supporting breast feeding; supporting healthy weight and healthy nutrition; improving health literacy, reducing accidents and minor illnesses; and supporting health, well-being and development: ready to learn and narrowing the ‘word gap’.^1^ It is hypothesised that more frequent health visiting contacts improve child outcomes within these high-impact areas, both at a universal level for all families^9 10^ and for specific groups of children.^1^ There is some evidence that intensive health visiting support (25–64 visits to a child’s home over 2 years by the same health visitor) improve some child outcomes for some families.^11–14^ However, there is no evidence on the impact of number or type of ‘usual service’ health visiting contacts on child outcomes, and scant evidence on costs and benefits.

In a challenging fiscal environment, with shrinking budgets and insufficient workforce, decisions need to be made about how to meet the increasing needs of families. The COVID-19 pandemic has exacerbated the situation, with redeployment of health visiting staff adding to the strain on services.^15^ As health visiting is a high-spend intervention provided by every local authority for every child under 5, there is huge potential to increase the impact and efficiency of the service through improved planning, commissioning and delivery. To achieve this, national policy-makers and local leaders need robust evidence on the benefits and costs of health visiting contacts, for different types of families in different local contexts.

This study aims to provide this evidence. It will integrate quantitative analysis of administrative data with qualitative case studies, estimating the extent to which different numbers and types of health visiting contacts improve child health and development and describing what is happening in health visiting contacts that ‘work’.

## Methods and analysis

### Aims, objectives and study design

The overall aim of the study is to answer the following research question: What is the effectiveness of health visiting in different contexts in England, how does it work, and at what cost?

The objectives are to:

1. Gather and review data and analysis on public health services for children aged 0–5 years, with a focus on identifying priority issues for professional and lay stakeholder groups.
2. Describe variation in number, type and timing of health visiting contacts across local authorities.
3. Use individual-level linked data to examine the association between number, type and timing of health visiting contacts and child/mother outcomes, including variation by child/mother/family characteristic and across local areas.
4. Use area-level data to examine the association between different local models of health visiting and child health and development outcomes, including variation by area-level characteristics.
5. Estimate the costs and benefits of health visiting services across local authorities with different types of health visiting service delivery models.
6. Generate an explanatory qualitative analysis of health visiting delivery (including during the COVID-19 pandemic) in selected case study sites that, in integration with other data, will contribute to a programme theory of health visiting.
7. Integrate the study components to produce robust, meaningful and useful findings for stakeholder groups: members of the public, policy, practice, academic.

This is a mixed-methods study with a sequential explanatory design.^16^ We use qualitative methods and substantial stakeholder input to explain quantitative analyses, test generalisability and causal inferences and interpret findings in the context of local policy, practice and circumstance.

### Quantitative data sources

#### Health, education and social care data (Education and Child Health Insights from Linked Data) linked to health visiting data (CSDS)

Our study will exploit national, individual-level health visiting activity data from the CSDS linked with data from children’s social care, hospitals and schools, captured within a new longitudinal dataset known as Education and Child Health Insights from Linked Data (ECHILD).^17^ CSDS includes information on individual health visiting contacts, including whether the appointment was ‘attended’ or ‘scheduled but not attended’, and whether it was a correspondence or face-to-face contact.^18^ ECHILD links data from hospital admissions, outpatient attendances, A&E and mortality (Hospital Episode Statistics (HES)), education (National Pupil Database) and children’s social care services (Child in Need and Looked after Children) for all children in England born from 1995.^17^ We will use ECHILD to create longitudinal trajectories for mother and child, capturing information on maternal education, maternal history of contact with children’s social care, maternal age at first birth, and number of siblings. Using these data, we will analyse health visiting activity, health and social care utilisation, and outcomes for all children in England aged under 5 and their mothers, from 2018 to 2019 onwards.

#### Health visiting taxonomy

As part of an existing study, we are developing a taxonomy of health visiting in all local authorities in England.^19^ The taxonomy will provide rich descriptions and explanations of 3–5 commonly used models of health visiting, for example: model 1—low universal coverage but highly targeted health visiting, conducted mostly by qualified health visitors; model 2—high universal coverage but few additional contacts, conducted mostly by band 5 nurses. This classification will be used as an exposure to estimate the association between local models of health visiting and area-level child health and development outcomes.

#### Aggregate local authority data

We will use Office for Health Improvement and Disparities Fingertips and Interim Reporting Statistics, and The Children’s Commissioner’s Vulnerability Profiles for aggregate area-level outcomes, where individual-level outcomes are not available (eg, on breast feeding or vaccination coverage).

### Analysis

#### Scoping

We will conduct a rapid review of literature on health visiting policy, practice and impact. We will gather additional insights through roundtables with policy-makers, interviews with practice and commissioning colleagues and workshops with parents. The key aims of these meetings are to (A) identify ’priority issues’ for different stakeholder groups, which will inform our research questions, analysis and outputs and (B) contribute to our theory of change underpinning the evaluation. The scoping review will ensure that our findings address the needs of policy-makers and professionals who commission, manage or deliver health visiting and reflect what is important to families.

#### Variation in health visiting contacts

We will use CSDS-ECHILD to describe the number, type (face to face, telephone; mandated or additional; home or clinic) and timing of health visiting contacts, and variation by local-area characteristics (eg, deprivation, rurality) and child/maternal characteristics (eg, birth order, singleton/multiple birth, number of siblings, maternal education, history of maternal adversity). We will focus our analysis on attended contacts, where the family sees a member of the health visiting team face to face. We will also examine ‘scheduled but not attended’ contacts to determine the extent to which differences in the number of attended contacts between different families or different local areas are due to ‘missed’ appointments or differences in administrative processes.

We will limit analyses to local authorities for which sufficiently complete health visiting activity data is captured in CSDS, using methods developed in a previous study.^7^ We will focus on health visiting services as delivered in financial years 2018/2019 and 2019/2020, as robust data on contacts during the COVID-19 pandemic is not available.

#### Impact of health visiting contacts

The impact of health visiting contacts will be assessed for children aged 0–5 years and their mothers at both individual and area level. The exposure for individual-level analysis will be the number, type and timing of health visiting contacts. At area level, the exposure will be provided by the local authority taxonomy of health visiting described above.

**Figure 1:**
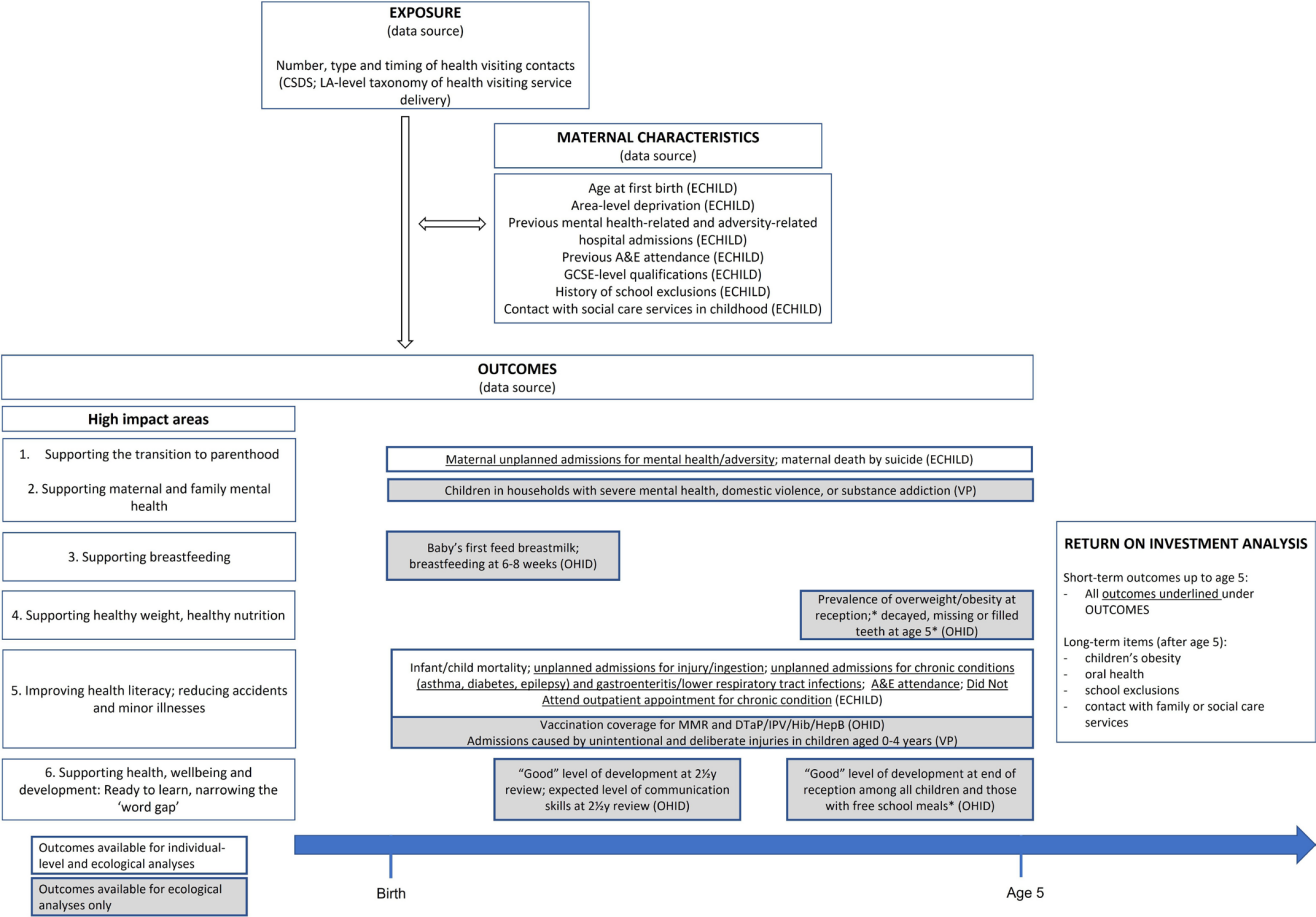
Overview of study outcomes. ECHILD includes linked data on hospital admissions, outpatient appointments and A&E attendance (Hospital Episode Statistics), mortality data (Office for National Statistics), educational data (National Pupil Database) and social care data (Child in Need and Looked after children datasets) for all people in England born from 1995. *These outcomes will be available, although measured on a subset of children receiving health visiting services before our exposure measure was defined (for 2018/2019); they, therefore, require the assumption that models of health visiting service delivery have not changed substantially in the few years before 2018/2019. They also might include children up to age 6. CSDS, Community Services Dataset; ECHILD, Education and Child Health Insights from Linked Data; OHID, Office for Health Improvement and Disparities Fingertips data; VP, Children’s Commissioner Vulnerability Profiles; GCSE, General Certificate of Secondary Education.

Outcomes will be defined based on the high-impact areas of the HCP (figure 1).

### High-impact areas 1 and 2: transition to parenthood, maternal and family mental health

Unplanned hospital admissions for mental health, substance misuse or violence; maternal death by suicide; 6–8 weeks postpartum GP check; timing of maternal mental health diagnosis and prescriptions; prevalence of children in households with severe mental health, domestic violence or substance addiction.

### High-impact area 3: supporting breast feeding

Breast feeding (6–8 weeks).

### High-impact area 4: supporting healthy weight, healthy nutrition

Prevalence of overweight/obesity (reception); decayed, missing or filled teeth (age 5).

### High-impact area 5: improving health literacy, reducing accidents and minor illnesses

Infant/child mortality; unplanned admissions for injury/ingestion; unplanned admissions for chronic conditions (asthma, diabetes, epilepsy) and gastroenteritis/lower respiratory tract infections; Accident & Emergency (A&E) attendance; did not attend outpatient appointment for chronic conditions; regular GP attendance for children with chronic conditions, including with nurse practitioners; vaccination coverage for measles, mumps and rubella (MMR) and diphtheria, tetanus, and pertussis (DTaP) / inactivated poliovirus vaccine (IPV) / Haemophilus influenzae type b (Hib) / Hepatitis type b (HepB).

### High-impact area 6: ready to learn and narrowing the word gap

‘Good’ level of development and expected communication skills at 2.5-year review; ‘good’ level of development at end of reception

A key challenge for analysing the association between the number of health visiting contacts and maternal/child outcomes is that families with higher need may be more likely to receive more intensive health visiting support. A simple comparison of outcomes for families receiving ‘high’ versus ‘low’ numbers of health visiting contacts could therefore produce associations between high levels of health visiting and poor outcomes (reverse causality).

To address this problem, we will use propensity score matching to minimise confounding due to differences between groups at baseline, by matching children with similar underlying need (according to maternal and child characteristics) but different numbers of health visiting contacts. This allows us to adjust for the propensity of families to receive different numbers or types of health visiting contacts.

One limitation of propensity scores is that we can only match families on known characteristics. In this study, we will have access to a large number of variables: child characteristics at birth (including gestational age, birth weight, ethnicity, area-level deprivation, number of siblings) and maternal characteristics before birth (including age at first birth, history of mental health-related hospital admissions, GCSE-level qualifications, mother’s contact with social care services in childhood). However, there may be some remaining confounding due to unmeasured factors such as income and parenting style.

The individual-level analysis will use multilevel regression models (logistic, Poisson or linear) to evaluate the relationship between the number and type of health visitor contacts and outcomes in the propensity-matched cohort, accounting for clustering within local authorities. Our primary analysis will be restricted to the earliest birth for each woman from 2018 to 2019 onwards to avoid statistical problems of clustering of children within mothers. To determine whether the effect of health visiting contacts is different for different groups, we will stratify by potential moderators at individual level (eg, birth order, singleton/multiple birth, ethnicity) and area level (eg, FNP, family hubs, deprivation, ethnic diversity).

In the area-level analysis, we will use the taxonomy described above to assign each local authority to a model of health visiting based on health visiting data from 2018 to 2019 and 2019 to 2020. We will create annual aggregated measures of outcomes for each local authority, for years 2018/2019–2021/2022. We will use linear mixed-effects models to analyse the relationship between health visiting model and outcomes, adjusting for area-level characteristics. This will allow us, for example, to evaluate the effect of model A vs model B of health visiting for children born in 2019 on the proportion of children with a good level of development at the 2–2.5 years review in 2021, in local authorities with similar levels of deprivation.

#### Return on investment analysis

The economic analysis will adhere to the National Institute for Health and Care Excellence methods and guidance for the evaluation of public health interventions.^20^ It will compare total costs to total benefits of different health visiting service delivery models within the local authority taxonomy described above.

The costs of health visiting services will be estimated using CSDS-ECHILD individual-level linked data on health visiting contacts (including duration), combined with unit costs. Unit costs will be obtained using data collected as part of an existing study,^19^ supplemented with costing information collected from the case study sites (see Case studies below). Where not available locally, unit costs for health visiting contacts will be obtained from National Health Service (NHS) reference costs.^21^

The main return on investment analysis will consider benefits in relation to outcomes for children aged 0–5 years. Outcomes related to contacts with the health system (eg, A&E attendance) or health outcomes with known long-term financial implications (eg, obesity) will be valued in monetary terms, using existing local data, information from the case study sites or national sources such as the Personal Social Services Research Unit costs of health and social care.^22^ To account for potential spillover effects, a secondary analysis will incorporate family and maternal health outcomes such as unplanned maternal hospital admissions for mental health.

We will use the results of these analyses and evidence from the literature to project longer-term benefits in relation to children’s obesity, oral health, school exclusions and contact with family or social care services. All outcomes included in the economic analysis are detailed in figure 1.

#### Case studies

Case studies will be conducted in up to four local authorities. Using Yin’s^23^ multiple case study design, we will analyse the process of health visiting service delivery in the selected locations, identifying mechanisms of change and determining best local practices that can be applied at a national level. Case study methodology emphasises the importance of context, including geography, demographic profile, local government, health services delivery and individual-level processes such as how health visitors make decisions on type or frequency of contact. This contextual understanding will help to explain why health visiting is delivered variably across England and why there may be different impacts for different groups of families and/or different local areas. Although COVID-19 is not a direct focus of the study, the qualitative analysis in these case studies will explore the impact of the pandemic on health visiting service delivery and contact types.

Sites will be selected to explain emerging findings from the impact analysis, while seeking maximum variation on population parameters including geography, demographic profile and local government. Data collection will include documentary analysis of local policy and protocols, in-depth interviews with health visiting practitioners, managers and commissioners, and focus groups or interviews with parents.

Interview topic guides will be developed using outputs of the previous stages of the analysis and our discussions with lay stakeholders. We will ask health visitors how they identify families at differing levels of need, and how decisions are made regarding the number, type, and timing of health visiting contacts, by asking about specific families on their case load.^24 25^ We will ask managers and commissioners how health visiting services are organised and delivered locally, and why this is the case. We will ask parents about their experiences and perceptions of health visiting and other relevant services, with prompts on ‘priority issues’ and important aspects of the theory of change identified in the scoping component. We will work with local stakeholders to gather data on the resources used to provide the health visiting service, which will inform the return on investment analysis.

#### Integration

**Figure 2:**
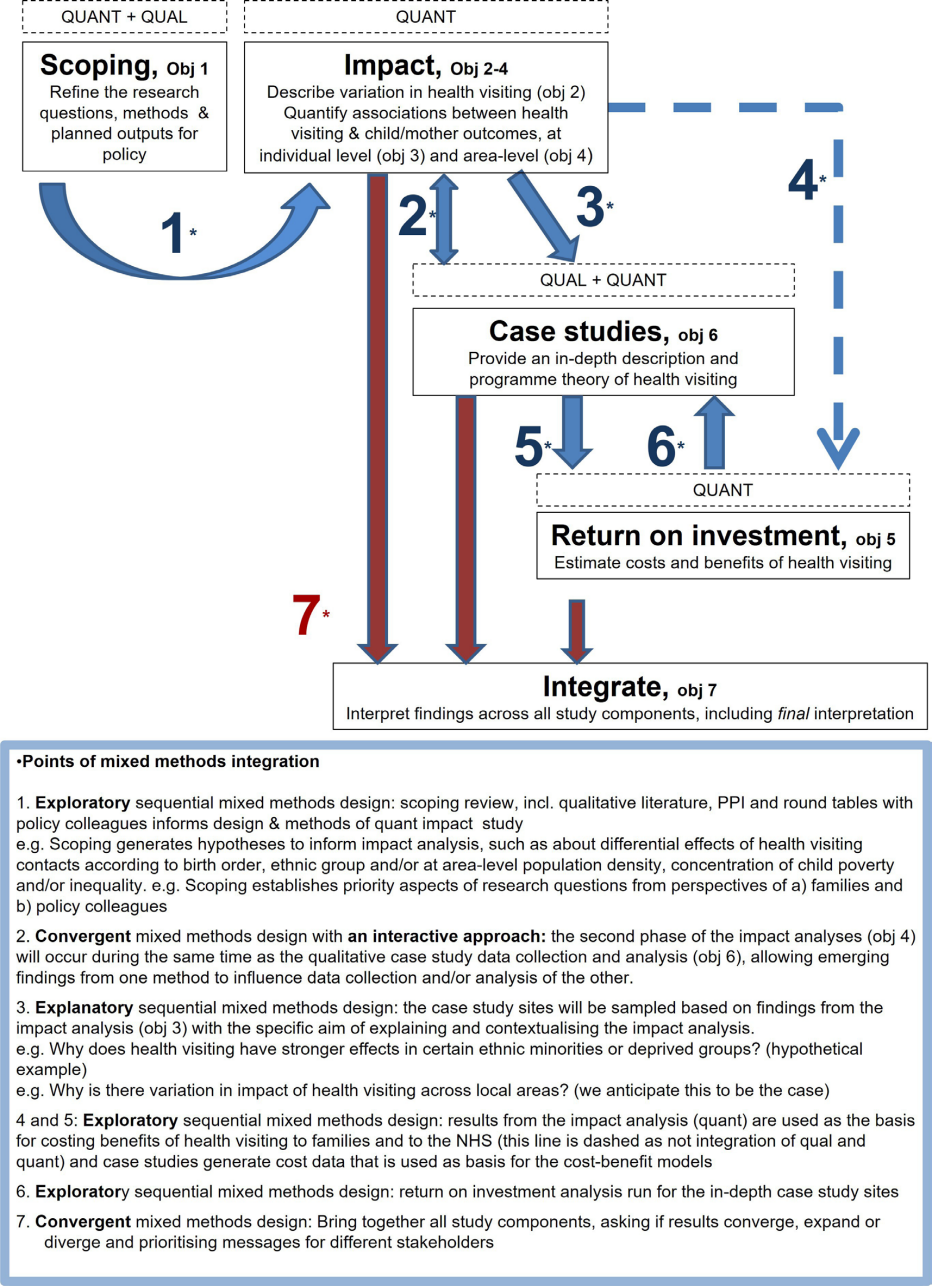
Integration of study components. NHS, National Health Service; PPI, patient and public involvement.

This complex, interdisciplinary study uses multiple methods to achieve its objectives (figure 2). It is widely recognised that integrating qualitative and quantitative data within a single study can bring unique insights, allowing more nuanced and deeper analysis of complex phenomena (such as health visiting) than studies using a single method.^26 27^ Achieving these benefits of a mixed-methods study relies on successfully integrating the data at the level of study design, analysis, interpretation and presentation.^28^ At study design level, we will adopt an ‘advanced multistage mixed-methods framework’^29^ combining three core approaches.

Exploratory sequential: The qualitative data collected through the scoping component will establish priority issues and generate hypotheses to pursue in the quantitative component and in the case studies.

Explanatory sequential: The qualitative interview data collected through case studies will help explain any variation in health visiting and the differential effects of health visiting that we find in the quantitative analyses.

Convergent: The area-level impact component will overlap with the case studies, and emerging findings will be tested for confirmation, expansion or divergence.

At the analysis and interpretation stages, we will take a ‘constant comparative approach’. This is a hallmark of grounded theory (a qualitative research method) but can be employed across data types and paradigms.^30^ We will hold regular ‘integration meetings’ where team members come together to compare challenges, insights and emerging findings across study components. All research outputs will draw on multiple components of the study, with priority issues and theory generated from the case studies playing a key role in integrating the findings throughout.

### Patient and public involvement

We have taken into account the views and experiences of parents when planning this study, via four patient and public involvement (PPI) sessions. Participants were aware that experiences of health visiting differed significantly between families and local areas and there was a strong message that reasons for variation should be understood. Parents highlighted how important it was to see the same health visitor (continuity of care) and to build a good relationship with ‘their’ health visitor. These concerns have informed our case study design, where we will conduct ‘deep dives’ into selected local areas to understand what drives variation, and the importance of the parent–professional relationship as a mechanism of effect.

For this study, we will establish three PPI groups: a virtual group of eight parents, and a group of mothers and a group of fathers from Barking and Dagenham in northeast London, with up to six participants in each. We will consult these groups at two key times, initially during the scoping review and towards the end of the study to help with interpretation and communication of findings.

## Ethics and dissemination

### Ethics

This study has been approved by University College London Research Ethics Committee (ref 20561/002) and the National Research Ethics Service (21/SW/0159). Access to ECHILD, HES and CSDS have been approved by NHS England (NIC-393510 and NIC-381972). The case study element involves NHS access and separate approval is being sought from the Health Research Authority.

### Dissemination

The findings of this study will be of benefit to national policy-makers, local directors of public health, commissioners and managers of health visiting services, health visitors and parents. Our research will inform national guidance on the health visiting component of the HCP, contributing to the modernisation of the HCP by the Department of Health and Social Care and the Office for Health Improvement and Disparities. Our findings will be used by local leaders as they develop and improve their Start for Life offer and their local HCP. Evidence about the impact, costs and benefits of health visiting services will inform submissions to the 2024 Spending Review.

We will produce policy briefings aimed at health visiting decision-makers and practitioners, and lay summaries, which can be used to raise awareness among parents of the purpose of health visiting and what they can expect from their contacts with health visitors. For researchers, we aim to publish our findings in peer-reviewed journal articles and present at key conferences.

## Supplementary Material

Reviewer comments

Author's manuscript
