# Supplementary material for: Study protocol: evaluation of the 0–5 public health investment in England – a mixed-methods study integrating analyses of national linked administrative data with in-depth case studies
Source: BMJ Open. 2023 Apr 5;13(4):e073313. doi: 10.1136/bmjopen-2023-073313 (PMC10083857; doi:10.1136/bmjopen-2023-073313)
Supplement: Author's manuscript [file bmjopen-2023-073313.draft_revisions.pdf]

BMJ Open is committed to open peer review. As part of this commitment we make the peer review history of every article we publish publicly available.

When an article is published we post the peer reviewers' comments and the authors' responses online. We also post the versions of the paper that were used during peer review. These are the versions that the peer review comments apply to.

The versions of the paper that follow are the versions that were submitted during the peer review process. They are not the versions of record or the final published versions. They should not be cited or distributed as the published version of this manuscript.

BMJ Open is an open access journal and the full, final, typeset and author-corrected version of record of the manuscript is available on our site with no access controls, subscription charges or pay-per-view fees (http://bmjopen.bmj.com).

If you have any questions on BMJ Open's open peer review process please email info.bmjopen@bmj.com

BMJ Open

Study protocol: evaluation of the 0-5 Public Health Investment in England: a mixed methods study integrating analyses of national linked administrative data with in-depth case studies

Journal:	*BMJ Open*
Manuscript ID	bmjopen-2023-073313
Article Type:	Protocol
Date Submitted by the Author:	02-Mar-2023

Complete List of Authors:	Harron, Katie; University College London, UCL Great Ormond Street Institute of Child Health<br>Cavallaro, Francesca; The Health Foundation<br>Bunting, Catherine; University College London, UCL Great Ormond Street Institute of Child Health<br>Clery, Amanda; University College London, UCL Great Ormond Street Institute of Child Health<br>Kendall, Sally; University of Kent, Centre for Health Services Studies<br>Cassidy, Rebecca; University of Kent, Centre for Health Services Studies<br>Atkins, Julie; Care City<br>Saloniki, Eirini-Christina; University College London, Department of Applied Health Research; NIHR Applied Research Collaboration North Thames<br>Bedford, Helen; University College London, UCL Great Ormond Street Institute of Child Health<br>Woodman, Jenny; UCL, Social Research Institute

Keywords:	Health policy < HEALTH SERVICES ADMINISTRATION & MANAGEMENT, Health economics < HEALTH SERVICES ADMINISTRATION & MANAGEMENT, Community child health < PAEDIATRICS, PUBLIC HEALTH

SCHOLARONE™
Manuscripts

Study protocol: evaluation of the 0-5 Public Health Investment in England: a mixed methods study integrating analyses of national linked administrative data with in-depth case studies

Authors: Katie Harron, Francesca L Cavallaro, Catherine Bunting, Amanda Clery, Sally Kendall, Rebecca Cassidy, Julie Atkins, Eirini-Christina Saloniki, Helen Bedford, Jenny Woodman

Correspondence to: Katie Harron, UCL GOS Institute of Child Health, 30 Guilford Street, London, WC1 N 1EH; k.harron@ucl.ac.uk

Word count: 3691, excluding abstract, footnotes, references

For peer review only

ABSTRACT

Introduction

Health visiting is a long-established, nationally implemented programme that works with other services at a local level to improve the health and wellbeing of children and families. To maximise the impact and efficiency of the health visiting programme, policy makers and commissioners need robust evidence on the costs and benefits of different levels and types of health visiting, for different families, in different local contexts.

Methods and analysis

This mixed-methods study will analyse individual-level health visiting data for 2018/19 and 2019/20 linked with longitudinal data from children's social care, hospitals and schools to estimate the association of number and type of health visiting contacts with a range of child and maternal outcomes. We will also use aggregate local authority data to estimate the association between local models of health visiting and area-level outcomes. Outcomes will include hospitalisations, breastfeeding, vaccination, childhood obesity and maternal mental health. Where possible, outcomes will be valued in monetary terms, and we will compare total costs to total benefits of different health visiting service delivery models. Qualitative case studies and extensive stakeholder input will help explain the quantitative analyses and interpret the results in the context of local policy, practice and circumstance.

Ethics and dissemination

The University College London Research Ethics Committee approved this study (ref 20561/002). Results will be submitted for publication in a peer-reviewed journal and findings will be shared and debated with national policy makers, commissioners and managers of health visiting services, health visitors and parents.

STRENGTHS AND LIMITATIONS OF THIS STUDY

- This interdisciplinary study will integrate data from multiple sources including regression analyses, return on investment analysis and qualitative case studies to provide a rich and nuanced account of the impact and value of health visiting in England.
- Extensive stakeholder input and public involvement will enable us to focus the research on priority issues for policymakers, practitioners and families, and to interpret the findings in the context of local area characteristics.
- Our analyses will use national data on health visiting (Community Services Dataset [CSDS]) linked to children's health, education and social care data (ECHILD), with longitudinal trajectories for mother and child. Since the CSDS is incomplete, our analyses will be restricted to areas for which sufficient data have been captured.
- We will use propensity score matching to compare families who have similar levels of underlying need but who receive different numbers or types of health visiting contact. Although this will increase our confidence that any observed differences in child and maternal outcomes are due to differences in the intensity of health visiting support received, families can only be matched on known characteristics, and there may be residual confounding due to unmeasured factors such as income or parenting style.

For peer review only - http://bmjopen.bmj.com/site/about/guidelines.xhtml

INTRODUCTION

Health visiting is a long-established, national intervention that works with other services including general practitioners (GPs), paediatricians and social care to maximise the health and wellbeing of children. Health visitors lead the Healthy Child Programme (HCP) for children aged 0-5 years in England, providing a universal, preventive service to families that includes health screening, immunisation, health and development reviews and parenting advice and support (1). The health visiting model is designed to be 'universal in reach – personalised in response' (1). It is underpinned by holistic needs assessment and relationship work with families, with health visitors offering guidance and interventions that are tailored to meet the needs of individual children and families (1, 2).

There are five universal mandated health visiting contacts: at 28 weeks pregnancy; 10-14 days after birth; 6-8 weeks; 9-12 months; and 2-2½ years. It is expected that most families will have most of their needs met by assessment, guidance and signposting within the five mandated contacts. Other families need more targeted support, including additional health visiting contacts and/or referral for interventions from other professionals such as early years, education, voluntary organisations, social services and GPs and primary care teams (1). Health visiting contacts can be face-to-face (at home or in a clinic or community centre), online, by telephone or by letter. Each contact may be delivered by the same or a different health visitor, or another member of the health visiting team such as a community staff nurse.

Health visiting service delivery varies across England, but little is known about the number or type of health visiting contacts that different families receive (3, 4). Public Health England statistics suggest that not all children receive their **mandated** reviews: coverage is lower for ethnic minorities and children living in deprived areas (5, 6). At the same time, two analyses using the Community Services Dataset (CSDS), the only national data on health visiting (5-7), suggest that children living in deprived areas receive more **additional** contacts (7, 8). These nuanced findings highlight the need for careful analysis of administrative data to describe routine health visiting, distinguishing between mandated and additional contacts and applying robust methods for handling missing data.

There is a lack of evidence on the impact of health visiting on child outcomes in England. Within the HCP, the six high impact areas for health visiting services are: supporting the transition to parenthood; supporting maternal and family mental health; supporting breast feeding; supporting healthy weight and healthy nutrition; improving health literacy, reducing accidents and minor illnesses; and supporting health, wellbeing and development: ready to learn and narrowing the 'word gap' (1). It is hypothesised that more frequent health visiting contacts improve child outcomes within these high impact areas, both at a universal level for all families (9, 10) and for specific groups of children (1). There is some evidence that intensive health visiting support (25-64 visits to a child's home over two years by the same health visitor) improve *some* child outcomes for *some* families (11-14). However, there is no evidence on the impact of number or type of 'usual service' health visiting contacts on child outcomes, and scant evidence on costs and benefits.

In a challenging fiscal environment, with shrinking budgets and insufficient workforce, decisions need to be made about how to meet the increasing needs of families. The COVID-19 pandemic has exacerbated the situation, with redeployment of health visiting staff adding to the strain on services (15). As health visiting is a high-spend intervention provided by every local authority for every child under 5, there is huge potential to increase the impact and efficiency of the service through improved planning, commissioning and delivery. To achieve this, national policy makers and local

For peer review only - http://bmjopen.bmj.com/site/about/guidelines.xhtml

leaders need robust evidence on the benefits and costs of health visiting contacts, for different types of families in different local contexts.

This study aims to provide this evidence. It will integrate quantitative analysis of administrative data with qualitative case studies, estimating the extent to which different numbers and types of health visiting contacts improve child health and development and describing what is happening in health visiting contacts that 'work'.

METHODS AND ANALYSIS

Aims, objectives and study design

The overall aim of the study is to answer the following research question: What is the effectiveness of health visiting in different contexts in England, how does it work, and at what cost?

The objectives are to:

1. Gather and review data and analysis on public health services for children aged 0-5 years, with a focus on identifying priority issues for professional and lay stakeholder groups

2. Describe variation in number, type and timing of health visiting contacts across local authorities

3. Use individual-level linked data to examine the association between number, type and timing of health visiting contacts and child/mother outcomes, including variation by child/mother/family characteristic and across local areas

4. Use area-level data to examine the association between different local models of health visiting and child health and development outcomes, including variation by area-level characteristics

5. Estimate the costs and benefits of health visiting services across local authorities with different types of health visiting service delivery models

6. Generate an explanatory qualitative analysis of health visiting delivery (including during the COVID-19 pandemic) in selected case study sites that, in integration with other data, will contribute to a programme theory of health visiting

7. Integrate the study components to produce robust, meaningful and useful findings for stakeholder groups: members of the public, policy, practice, academic

This is a mixed methods study with a sequential explanatory design (16). We use qualitative methods and substantial stakeholder input to explain quantitative analyses, test generalisability and causal inferences and interpret findings in the context of local policy, practice and circumstance.

Quantitative data sources

Health, education and social care data (ECHILD) linked to health visiting data (CSDS)

Our study will exploit national, individual-level health visiting activity data from the CSDS linked with data from children's social care, hospitals and schools, captured within a new longitudinal dataset known as ECHILD (17). CSDS includes information on individual health visiting contacts, including

whether the appointment was 'attended' or 'scheduled but not attended', and whether it was a correspondence or face-to-face contact (18). ECHILD links data from hospital admissions, outpatient attendances, A&E and mortality (Hospital Episode Statistics [HES]), education (National Pupil Database) and children's social care services (Child in Need and Looked after Children) for all children in England born from 1995 (17). We will use ECHILD to create longitudinal trajectories for mother and child, capturing information on maternal education, maternal history of contact with children's social care, maternal age at first birth, and number of siblings. Using these data, we will analyse health visiting activity, health and social care utilisation, and outcomes for all children in England aged under five and their mothers, from 2018/19 onwards.

Health visiting taxonomy

As part of an existing study, we are developing a taxonomy of health visiting in all local authorities in England (19). The taxonomy will provide rich descriptions and explanations of 3-5 commonly used models of health visiting, for example: Model 1 – low universal coverage but highly targeted health visiting, conducted mostly by qualified health visitors; Model 2 – high universal coverage but few additional contacts, conducted mostly by band 5 nurses. This classification will be used as an exposure to estimate the association between local models of health visiting and area-level child health and development outcomes.

Aggregate local authority data

We will use Office for Health Improvement & Disparities Fingertips and Interim Reporting Statistics, and The Children's Commissioner's Vulnerability Profiles for aggregate area-level outcomes, where individual-level outcomes are not available (for example, on breastfeeding or vaccination coverage).

Analysis

Scoping

We will conduct a rapid review of literature on health visiting policy, practice and impact. We will gather additional insights through roundtables with policymakers, interviews with practice and commissioning colleagues and workshops with parents. The key aims of these meetings are to a) identify 'priority issues' for different stakeholder groups, which will inform our research questions, analysis and outputs; and b) contribute to our theory of change underpinning the evaluation. The scoping review will ensure that our findings address the needs of policymakers and professionals who commission, manage or deliver health visiting, and reflect what is important to families.

Variation in health visiting contacts

We will use CSDS-ECHILD to describe the number, type (face-to-face, telephone; mandated or additional; home or clinic) and timing of health visiting contacts, and variation by local-area characteristics (e.g. deprivation, rurality) and child/maternal characteristics (e.g. birth order, singleton/multiple birth, number of siblings, maternal education, history of maternal adversity). We will focus our analysis on attended contacts, where the family sees a member of the health visiting team face-to-face. We will also examine 'scheduled but not attended' contacts to determine the extent to which differences in the number of attended contacts between different families or different local areas are due to 'missed' appointments or differences in administrative processes.

We will limit analyses to local authorities for which sufficiently complete health visiting activity data is captured in CSDS, using methods developed in a previous study (7). We will focus on health visiting services as delivered in financial years 2018/19 and 2019/20, as robust data on contacts during the COVID-19 pandemic is not available.

For peer review only

Impact of health visiting contacts

The impact of health visiting contacts will be assessed for children aged 0-5 years and their mothers at both individual and area level. The exposure for individual-level analysis will be number, type and timing of health visiting contacts. At area level, the exposure will be provided by the local authority taxonomy of health visiting described above.

Outcomes will be defined based on the high impact areas of the HCP (Figure 1):

High impact areas 1&2: Transition to parenthood, maternal and family mental health
Unplanned hospital admissions for mental health, substance misuse or violence; maternal death by suicide; 6-8 week postpartum GP check; timing of maternal mental health diagnosis and prescriptions; prevalence of children in households with severe mental health, domestic violence, or substance addiction

High impact area 3: Supporting breastfeeding
Breastfeeding (6-8 weeks)

High impact area 4: Supporting healthy weight, healthy nutrition
Prevalence of overweight/obesity (reception); decayed, missing or filled teeth (age 5)

High impact area 5: Improving health literacy, reducing accidents and minor illnesses
Infant/child mortality; unplanned admissions for injury/ingestion; unplanned admissions for chronic conditions (asthma, diabetes, epilepsy) and gastroenteritis/lower respiratory tract infections; A&E attendance; Did Not Attend outpatient appointment for chronic conditions; regular GP attendance for children with chronic conditions, including with nurse practitioners; vaccination coverage for MMR and DTaP/IPV/Hib/HepB

High impact area 6: Ready to learn and narrowing the word gap
"Good" level of development and expected communication skills at 2½ year review; "Good" level of development at end of reception

A key challenge for analysing the association between number of health visiting contacts and maternal/child outcomes is that families with higher need may be more likely to receive more intensive health visiting support. A simple comparison of outcomes for families receiving 'high' versus 'low' numbers of health visiting contacts could therefore produce associations between high levels of health visiting and poor outcomes (reverse causality).

To address this problem, we will use propensity score matching to minimise confounding due to differences between groups at baseline, by matching children with similar underlying need (according to maternal and child characteristics) but different numbers of health visiting contacts. This allows us to adjust for the *propensity* of families to receive different numbers or types of health visiting contacts.

One limitation of propensity scores is that we can only match families on known characteristics. In this study, we will have access to a large number of variables: child characteristics at birth (including gestational age, birthweight, ethnicity, area-level deprivation, number of siblings) and maternal characteristics before birth (including age at first birth, history of mental health-related hospital admissions, GCSE-level qualifications, mother's contact with social care services in childhood). However, there may be some remaining confounding due to unmeasured factors such as income and parenting style.

For peer review only

The individual-level analysis will use multi-level regression models (logistic, Poisson or linear) to evaluate the relationship between number and type of health visitor contacts and outcomes in the propensity-matched cohort, accounting for clustering within local authorities. Our primary analysis will be restricted to the earliest birth for each woman from 2018/19 onwards to avoid statistical problems of clustering of children within mothers. To determine whether the effect of health visiting contacts is different for different groups, we will stratify by potential moderators at individual level (e.g. birth order, singleton/multiple birth, ethnicity) and area level (e.g. FNP, Family Hubs, deprivation, ethnic diversity).

In the area-level analysis we will use the taxonomy described above to assign each local authority to a model of health visiting based on health visiting data from 2018/19 and 2019/20. We will create annual aggregated measures of outcomes for each local authority, for years 2018/19-2021/22. We will use linear mixed-effects models to analyse the relationship between health visiting model and outcomes, adjusting for area-level characteristics. This will allow us, for example, to evaluate the effect of model A versus model B of health visiting for children born in 2019 on the proportion of children with a good level of development at the 2-2½ year review in 2021, in local authorities with similar levels of deprivation.

Return on investment analysis

The economic analysis will adhere to the National Institute for Health and Care Excellence (NICE) methods and guidance for the evaluation of public health interventions (20). It will compare total costs to total benefits of different health visiting service delivery models within the local authority taxonomy described above.

The costs of health visiting services will be estimated using CSDS-ECHILD individual-level linked data on health visiting contacts (including duration), combined with unit costs. Unit costs will be obtained using data collected as part of an existing study (19), supplemented with costing information collected from the case study sites (see below). Where not available locally, unit costs for health visiting contacts will be obtained from NHS reference costs (21).

The main return on investment analysis will consider benefits in relation to outcomes for children aged 0-5 years. Outcomes related to contacts with the health system (e.g. A&E attendance) or health outcomes with known long-term financial implications (e.g. obesity) will be valued in monetary terms, using existing local data, information from the case study sites or national sources such as the Personal Social Services Research Unit costs of health and social care (22). To account for potential spillover effects, a secondary analysis will incorporate family and maternal health outcomes such as unplanned maternal hospital admissions for mental health.

We will use the results of these analyses and evidence from the literature to project longer-term benefits in relation to children's obesity, oral health, school exclusions and contact with family or social care services. All outcomes included in the economic analysis are detailed in Figure 1.

Case studies

Case studies will be conducted in up to four local authorities. Using Yin's (23) multiple case study design we will analyse the process of health visiting service delivery in the selected locations, identifying mechanisms of change and determining best local practices that can be applied at a national level. Case study methodology emphasises the importance of context, including geography, demographic profile, local government, health services delivery and individual-level processes such as how health visitors make decisions on type or frequency of contact. This contextual understanding will help to explain why health visiting is delivered variably across England and why there may be different impacts for different groups of families and/or different local areas. Although

For peer review only - http://bmjopen.bmj.com/site/about/guidelines.xhtml

COVID-19 is not a direct focus of the study, the qualitative analysis in these case studies will explore the impact of the pandemic on health visiting service delivery and contact types.

Sites will be selected to explain emerging findings from the impact analysis, while seeking maximum variation on population parameters including geography, demographic profile and local government. Data collection will include documentary analysis of local policy and protocols, in-depth interviews with health visiting practitioners, managers and commissioners, and focus groups or interviews with parents.

Interview topic guides will be developed using outputs of the previous stages of the analysis and our discussions with lay stakeholders. We will ask health visitors how they identify families at differing levels of need, and how decisions are made regarding the number, type, and timing of health visiting contacts, by asking about specific families on their case-load (24, 25). We will ask managers and commissioners how health visiting services are organised and delivered locally, and why this is the case. We will ask parents about their experiences and perceptions of health visiting and other relevant services, with prompts on 'priority issues' and important aspects of the theory of change identified in the scoping component. We will work with local stakeholders to gather data on the resources used to provide the health visiting service, which will inform the return on investment analysis.

Integration

This complex, interdisciplinary study uses multiple methods to achieve its objectives (Figure 2). It is widely recognised that integrating qualitative and quantitative data within a single study can bring unique insights, allowing more nuanced and deeper analysis of complex phenomena (such as health visiting) than studies using a single method (26, 27). Achieving these benefits of a mixed methods study relies on successfully integrating the data at the level of study design, analysis, interpretation and presentation (28). At study design level, we will adopt an 'advanced multistage mixed methods framework' (29) combining three core approaches:

Exploratory sequential: The qualitative data collected through the scoping component will establish priority issues and generate hypotheses to pursue in the quantitative component and in the case studies.

Explanatory sequential: The qualitative interview data collected through case studies will help explain any variation in health visiting and the differential effects of health visiting that we find in the quantitative analyses

Convergent: The area-level impact component will overlap with the case studies, and emerging findings will be tested for confirmation, expansion or divergence.

At analysis and interpretation stages, we will take a 'constant comparative approach'. This is a hallmark of Grounded Theory (a qualitative research method) but can be employed across data types and paradigms (30). We will hold regular 'integration meetings' where team members come together to compare challenges, insights and emerging findings across study components. All research outputs will draw on multiple components of the study, with priority issues and theory generated from the case studies playing a key role in integrating the findings throughout.

Patient and Public Involvement

We have taken into account the views and experiences of parents when planning this study, via four PPI sessions. Participants were aware that experiences of health visiting differed significantly

For peer review only

between families and local areas and there was a strong message that reasons for variation should be understood. Parents highlighted how important it was to see the same health visitor (continuity of care) and to build a good relationship with 'their' health visitor. These concerns have informed our case study design, where we will conduct ‘deep dives’ into selected local areas to understand what drives variation, and the importance of the parent-professional relationship as a mechanism of effect.

For this study we will establish three PPI groups: a virtual group of eight parents, and a group of mothers and a group of fathers from Barking and Dagenham in northeast London, with up to six participants in each. We will consult these groups at two key times, initially during the scoping review and towards the end of the study to help with interpretation and communication of findings.

ETHICS AND DISSEMINATION

Ethics

This study has been approved by University College London Research Ethics Committee (ref 20561/002) and the National Research Ethics Service (21/SW/0159). Access to ECHILD, HES and CSDS has been approved by NHS England (NIC-393510 and NIC-381972). The case study element involves NHS access and separate approval is being sought from the Health Research Authority.

Dissemination

The findings of this study will be of benefit to national policy makers, local directors of public health, commissioners and managers of health visiting services, health visitors and parents. Our research will inform national guidance on the health visiting component of the HCP, contributing to the modernisation of the HCP by the Department of Health and Social Care and the Office for Health Improvement and Disparities. Our findings will be used by local leaders as they develop and improve their Start for Life offer and their local HCP. Evidence about the impact, costs and benefits of health visiting services will inform submissions to the 2024 Spending Review.

We will produce policy briefings aimed at health visiting decision-makers and practitioners, and lay summaries, which can be used to raise awareness among parents of the purpose of health visiting and what they can expect from their contacts with health visitors. For researchers, we aim to publish our findings in peer-reviewed journal articles and present at key conferences.

For peer review only

FOOTNOTES

Funding: This work is supported by the National Institute for Health Research (NIHR) Health Services Policy Research Programme (NIHR203450). This research was supported in part by the NIHR Great Ormond Street Hospital Biomedical Research Centre.

Disclaimer: The views expressed are those of the author(s) and not necessarily those of the NIHR or the Department of Health and Social Care.

Competing interests: None declared

Patient and public involvement: Patients and/or the public were involved in the design, or conduct, or reporting, or dissemination plans of this research. Refer to the Methods section for further details.

Provenance and peer review: Not commissioned; peer reviewed for ethical and funding approval prior to submission.

For peer review only

REFERENCES

1. Public Health England. Health visiting and school nursing service delivery model - Guidance 2021 [Available from: https://www.gov.uk/government/publications/commissioning-of-public-health-services-for-children/health-visiting-and-school-nursing-service-delivery-model.
2. Woodman J, Simon A, Hauari H, Gilbert R. A scoping review of 'think-family' approaches in healthcare settings. Journal of public health (Oxford, England). 2020;42(1):21-37.
3. Public Health England. Rapid review to update evidence for the Healthy Child Programme 0-5. London; 2015.
4. Asmussen K, Feinstein L, Martin J, Chowdry H. Foundations for life: What works to support parent child interaction in the early years. London: EIF; 2016.
5. Public Health England. Health visitor service delivery metrics: 2018/19 annual data statistical commentary. 2019.
6. Public Health England. Characteristics of children receiving universal health visitor reviews 2021 [Available from: https://www.gov.uk/government/publications/characteristics-of-children-receiving-universal-health-visitor-reviews.
7. Fraser C, Harron K, Barlow J, Bennett S, Woods G, Shand J, et al. How can we use the community services dataset (CSDS) for research into health visiting? 2020 [Available from: https://www.ucl.ac.uk/children-policy-research/sites/children-policy-research/files/using_csds_for_research_report_08.10.20.pdf.
8. Public Health England. Contacts other than those mandated and referrals by health visitors - Experimental analysis of the Community Services Dataset. 2021.
9. Black M, Barnes A, Baxter S, Beynon C, Clowes M, Dallat M, et al. Learning across the UK: a review of public health systems and policy approaches to early child development since political devolution. Journal of Public Health. 2019;42(2):224-38.
10. Institute of Health Visiting. Health Visiting in England: A Vision for the Future 2019 [Available from: https://ihv.org.uk/wp-content/uploads/2019/11/7.11.19-Health-Visiting-in-England-Vision-FINAL-VERSION.pdf.
11. Goldfeld S, Bryson H, Mensah F, Gold L, Orsini F, Perlen S, et al. Nurse Home Visiting and Maternal Mental Health: 3-Year Follow-Up of a Randomized Trial. Pediatrics. 2021;147(2):e2020025361.
12. Asmussen K, Brims L. What works to enhance the effectiveness of the Healthy Child Programme: An Evidence Update. London; 2018.
13. Kemp L, Harris E, McMahon C, Matthey S, Vimpani G, Anderson T, et al. Child and family outcomes of a long-term nurse home visitation programme: a randomised controlled trial. Archives of Disease in Childhood. 2011;96(6):533-40.
14. Cavallaro F, Gilbert R, van der Meulen J, Kendall S, Kennedy E, Harron K. Evaluation of Intensive Home Visiting for Adolescent Mothers in the Family Nurse Partnership: A Population Based-Data Linkage Cohort Study in England. Available from: https://ssrn.com/abstract=4337372.
15. Conti G, Dow A. Using FOI data to assess the state of Health Visiting Services in England before and during COVID-19 London: UCL Department of Economics; 2021 [Available from: https://discovery.ucl.ac.uk/id/eprint/10132710/1/Conti_Dow_COVID-19_Health_Visiting_FOI1_POSTING_Aug.2021.pdf.
16. Tariq S, Woodman J. Using mixed methods in health research. JRSM Short Reports. 2013;4(6):2042533313479197.
17. Mc Grath-Lone L, Blackburn R, Gilbert R. The Education and Child Health Insights from Linked Data (ECHILD) Database: An Introductory Guide for Researchers 2021 [Available from: https://www.ucl.ac.uk/child-health/sites/child-health/files/echild_user_guide_v1.1.0.pdf.

For peer review only

18. Fraser C, Harron K, Barlow J, Bennett S, Woods G, Shand J, et al. Variation in health visiting contacts for children in England: cross-sectional analysis of the 2-2(1/2) year review using administrative data (Community Services Dataset, CSDS). BMJ Open. 2022;12(2):e053884.
19. Woodman J, Mc Grath-Lone L, Clery A, Weatherly H, Jankovic D, Appleton JV, et al. Study protocol: a mixed-methods study to evaluate which health visiting models in England are most promising for mitigating the harms of adverse childhood experiences. BMJ Open. 2022;12:e066880.
20. National Institute for Health and Care Excellence. Methods for the Development of NICE Public Health Guidance London: National Institute for Health and Care Excellence (NICE). Process and Methods Guides No. 4; 2012 [Available from: https://www.nice.org.uk/process/pmg4/chapter/incorporating-health-economics
21. Department of Health and Social Care. National Schedule of Reference Costs Year 2018–2019 London: Department of Health and Social Care; 2018 [Available from: https://www.england.nhs.uk/publication/2018-19-national-cost-collection-data-publication/
22. Curtis L, Burns A. Unit Costs of Health and Social Care 2018: Personal Social Services and Research Unit, University of Kent, Canterbury; 2018.
23. Yin R. Case study research and applications: Design and methods. Sixth edition: Sage publications; 2018.
24. Public Health England. Best start in life and beyond: improving public health outcomes for children, young people and families: guidance to support the commissioning of the Health Child Programme 0-19: Health Visiting and school nursing services. Commissioning guide 1: background information on comissioning and service model. London; 2018.
25. Department for Health. Healthy Child Programme. Pregnancy and the First Five Years. London; 2009.
26. O'Cathain A, Murphy E, Nicholl J. Three techniques for integrating data in mixed methods studies. BMJ. 2010;341:c4587.
27. Castro FG, Kellison JG, Boyd SJ, Kopak A. A Methodology for Conducting Integrative Mixed Methods Research and Data Analyses. J Mix Methods Res. 2010;4(4):342-60.
28. Guetterman TC, Fetters MD, Creswell JW. Integrating Quantitative and Qualitative Results in Health Science Mixed Methods Research Through Joint Displays. Ann Fam Med. 2015;13(6):554-61.
29. Fetters MD, Curry LA, Creswell JW. Achieving integration in mixed methods designs-principles and practices. Health services research. 2013;48(6 Pt 2):2134-56.
30. Mathison Se. Constant Comparative Method. Encyclopedia of Evaluation. Thousand Oaks, California2005.

Figure 1 Overview of study outcomes

Figure 2 Integration of study components

For peer review only

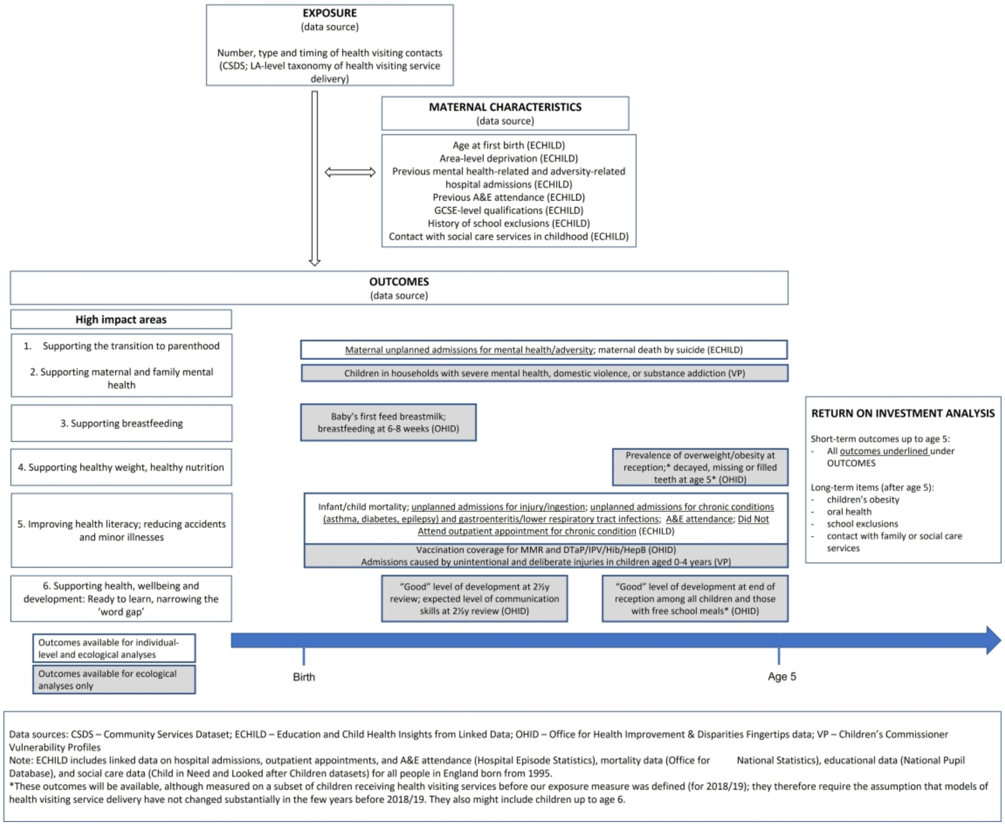

Figure 1. Overview of study outcomes

488x400mm (300 x 300 DPI)

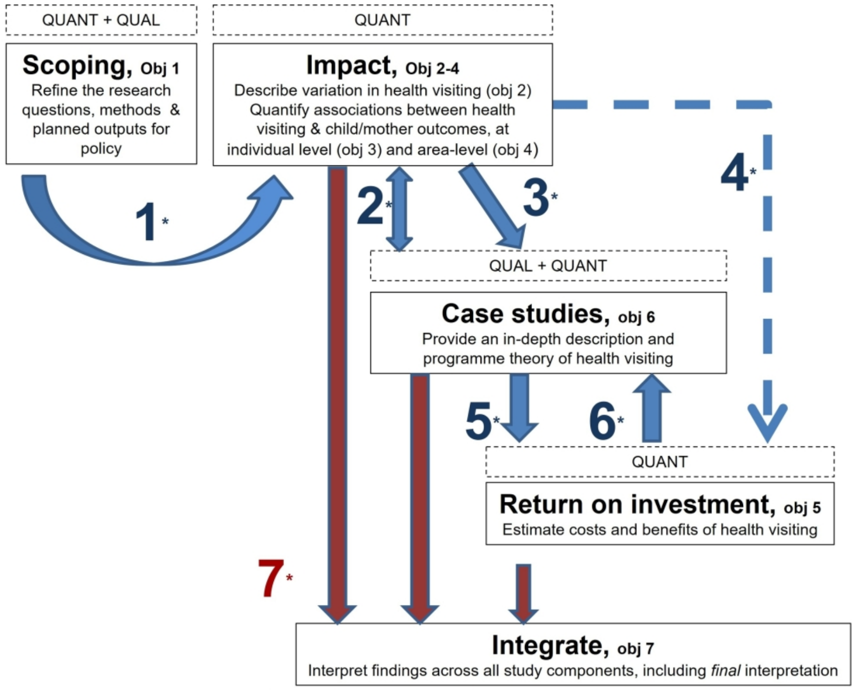

•**Points of mixed methods integration**

1. **Exploratory** sequential mixed methods design: scoping review, incl. qualitative literature, PPI and round tables with policy colleagues informs design & methods of quant impact study
e.g. Scoping generates hypotheses to inform impact analysis, such as about differential effects of health visiting contacts according to birth order, ethnic group and/or at area-level population density, concentration of child poverty and/or inequality. e.g. Scoping establishes priority aspects of research questions from perspectives of a) families and b) policy colleagues

2. **Convergent** mixed methods design with **an interactive approach:** the second phase of the impact analyses (obj 4) will occur during the same time as the qualitative case study data collection and analysis (obj 6), allowing emerging findings from one method to influence data collection and/or analysis of the other.

3. **Explanatory** sequential mixed methods design: the case study sites will be sampled based on findings from the impact analysis (obj 3) with the specific aim of explaining and contextualising the impact analysis.
e.g. Why does health visiting have stronger effects in certain ethnic minorities or deprived groups? (hypothetical example)
e.g. Why is there variation in impact of health visiting across local areas? (we anticipate this to be the case)

4 and 5: **Exploratory** sequential mixed methods design: results from the impact analysis (quant) are used as the basis for costing benefits of health visiting to families and to the NHS (this line is dashed as not integration of qual and quant) and case studies generate cost data that is used as basis for the cost-benefit models

6. **Explorator**y sequential mixed methods design: return on investment analysis run for the in-depth case study sites

7. **Convergent** mixed methods design: Bring together all study components, asking if results converge, expand or diverge and prioritising messages for different stakeholders

Figure 2. Integration of study components

210x289mm (300 x 300 DPI)

BMJ Open

Study protocol: evaluation of the 0-5 Public Health Investment in England: a mixed methods study integrating analyses of national linked administrative data with in-depth case studies

Journal:	*BMJ Open*
Manuscript ID	bmjopen-2023-073313.R1
Article Type:	Protocol
Date Submitted by the Author:	09-Mar-2023
Complete List of Authors:	Harron, Katie; University College London, UCL Great Ormond Street Institute of Child Health<br>Cavallaro, Francesca; The Health Foundation<br>Bunting, Catherine; University College London, UCL Great Ormond Street Institute of Child Health<br>Clery, Amanda; University College London, UCL Great Ormond Street Institute of Child Health<br>Kendall, Sally; University of Kent, Centre for Health Services Studies<br>Cassidy, Rebecca; University of Kent, Centre for Health Services Studies<br>Atkins, Julie; Care City<br>Saloniki, Eirini-Christina; University College London, Department of Applied Health Research; NIHR Applied Research Collaboration North Thames<br>Bedford, Helen; University College London, UCL Great Ormond Street Institute of Child Health<br>Woodman, Jenny; UCL, Social Research Institute
**Primary Subject Heading**:	Public health
Secondary Subject Heading:	Health economics, Health policy, Nursing
Keywords:	Health policy < HEALTH SERVICES ADMINISTRATION & MANAGEMENT, Health economics < HEALTH SERVICES ADMINISTRATION & MANAGEMENT, Community child health < PAEDIATRICS, PUBLIC HEALTH

SCHOLARONE™
Manuscripts

Study protocol: evaluation of the 0-5 Public Health Investment in England: a mixed methods study integrating analyses of national linked administrative data with in-depth case studies

Authors: Katie Harron, Francesca L Cavallaro, Catherine Bunting, Amanda Clery, Sally Kendall, Rebecca Cassidy, Julie Atkins, Eirini-Christina Saloniki, Helen Bedford, Jenny Woodman

Correspondence to: Katie Harron, UCL GOS Institute of Child Health, 30 Guilford Street, London, WC1 N 1EH; k.harron@ucl.ac.uk

Word count: 3641, excluding abstract, footnotes, references

For peer review only

ABSTRACT

Introduction
Health visiting is a long-established, nationally implemented programme that works with other services at a local level to improve the health and wellbeing of children and families. To maximise the impact and efficiency of the health visiting programme, policy makers and commissioners need robust evidence on the costs and benefits of different levels and types of health visiting, for different families, in different local contexts.

Methods and analysis
This mixed-methods study will analyse individual-level health visiting data for 2018/19 and 2019/20 linked with longitudinal data from children's social care, hospitals and schools to estimate the association of number and type of health visiting contacts with a range of child and maternal outcomes. We will also use aggregate local authority data to estimate the association between local models of health visiting and area-level outcomes. Outcomes will include hospitalisations, breastfeeding, vaccination, childhood obesity and maternal mental health. Where possible, outcomes will be valued in monetary terms, and we will compare total costs to total benefits of different health visiting service delivery models. Qualitative case studies and extensive stakeholder input will help explain the quantitative analyses and interpret the results in the context of local policy, practice and circumstance.

Ethics and dissemination
The University College London Research Ethics Committee approved this study (ref 20561/002). Results will be submitted for publication in a peer-reviewed journal and findings will be shared and debated with national policy makers, commissioners and managers of health visiting services, health visitors and parents.

STRENGTHS AND LIMITATIONS OF THIS STUDY

- This interdisciplinary study will integrate data from multiple sources including regression analyses, return on investment analysis and qualitative case studies to provide a rich and nuanced account of the impact and value of health visiting in England.
- Extensive stakeholder input and public involvement will enable us to focus the research on priority issues for policymakers, practitioners and families, and to interpret the findings in the context of local area characteristics.
- Our analyses will use data on health visiting from the Community Services Dataset (CSDS), restricted to areas for which sufficient data have been captured and linked to children's health, education and social care data (ECHILD).
- We will use propensity score matching to compare the outcomes of families with similar levels of underlying need who receive different levels of health visiting support, but there may be residual confounding due to unmeasured factors such as income or parenting style.

For peer review only - http://bmjopen.bmj.com/site/about/guidelines.xhtml

INTRODUCTION

Health visiting is a long-established, national intervention that works with other services including general practitioners (GPs), paediatricians and social care to maximise the health and wellbeing of children. Health visitors lead the Healthy Child Programme (HCP) for children aged 0-5 years in England, providing a universal, preventive service to families that includes health screening, immunisation, health and development reviews and parenting advice and support (1). The health visiting model is designed to be 'universal in reach – personalised in response' (1). It is underpinned by holistic needs assessment and relationship work with families, with health visitors offering guidance and interventions that are tailored to meet the needs of individual children and families (1, 2).

There are five universal mandated health visiting contacts: at 28 weeks pregnancy; 10-14 days after birth; 6-8 weeks; 9-12 months; and 2-2½ years. It is expected that most families will have most of their needs met by assessment, guidance and signposting within the five mandated contacts. Other families need more targeted support, including additional health visiting contacts and/or referral for interventions from other professionals such as early years, education, voluntary organisations, social services and GPs and primary care teams (1). Health visiting contacts can be face-to-face (at home or in a clinic or community centre), online, by telephone or by letter. Each contact may be delivered by the same or a different health visitor, or another member of the health visiting team such as a community staff nurse.

Health visiting service delivery varies across England, but little is known about the number or type of health visiting contacts that different families receive (3, 4). Public Health England statistics suggest that not all children receive their **mandated** reviews: coverage is lower for ethnic minorities and children living in deprived areas (5, 6). At the same time, two analyses using the Community Services Dataset (CSDS), the only national data on health visiting (5-7), suggest that children living in deprived areas receive more **additional** contacts (7, 8). These nuanced findings highlight the need for careful analysis of administrative data to describe routine health visiting, distinguishing between mandated and additional contacts and applying robust methods for handling missing data.

There is a lack of evidence on the impact of health visiting on child outcomes in England. Within the HCP, the six high impact areas for health visiting services are: supporting the transition to parenthood; supporting maternal and family mental health; supporting breast feeding; supporting healthy weight and healthy nutrition; improving health literacy, reducing accidents and minor illnesses; and supporting health, wellbeing and development: ready to learn and narrowing the 'word gap' (1). It is hypothesised that more frequent health visiting contacts improve child outcomes within these high impact areas, both at a universal level for all families (9, 10) and for specific groups of children (1). There is some evidence that intensive health visiting support (25-64 visits to a child's home over two years by the same health visitor) improve *some* child outcomes for *some* families (11-14). However, there is no evidence on the impact of number or type of 'usual service' health visiting contacts on child outcomes, and scant evidence on costs and benefits.

In a challenging fiscal environment, with shrinking budgets and insufficient workforce, decisions need to be made about how to meet the increasing needs of families. The COVID-19 pandemic has exacerbated the situation, with redeployment of health visiting staff adding to the strain on services (15). As health visiting is a high-spend intervention provided by every local authority for every child under 5, there is huge potential to increase the impact and efficiency of the service through improved planning, commissioning and delivery. To achieve this, national policy makers and local

For peer review only - http://bmjopen.bmj.com/site/about/guidelines.xhtml

leaders need robust evidence on the benefits and costs of health visiting contacts, for different types of families in different local contexts.

This study aims to provide this evidence. It will integrate quantitative analysis of administrative data with qualitative case studies, estimating the extent to which different numbers and types of health visiting contacts improve child health and development and describing what is happening in health visiting contacts that ‘work’.

METHODS AND ANALYSIS

Aims, objectives and study design

The overall aim of the study is to answer the following research question: What is the effectiveness of health visiting in different contexts in England, how does it work, and at what cost?

The objectives are to:

1. Gather and review data and analysis on public health services for children aged 0-5 years, with a focus on identifying priority issues for professional and lay stakeholder groups

2. Describe variation in number, type and timing of health visiting contacts across local authorities

3. Use individual-level linked data to examine the association between number, type and timing of health visiting contacts and child/mother outcomes, including variation by child/mother/family characteristic and across local areas

4. Use area-level data to examine the association between different local models of health visiting and child health and development outcomes, including variation by area-level characteristics

5. Estimate the costs and benefits of health visiting services across local authorities with different types of health visiting service delivery models

6. Generate an explanatory qualitative analysis of health visiting delivery (including during the COVID-19 pandemic) in selected case study sites that, in integration with other data, will contribute to a programme theory of health visiting

7. Integrate the study components to produce robust, meaningful and useful findings for stakeholder groups: members of the public, policy, practice, academic

This is a mixed methods study with a sequential explanatory design (16). We use qualitative methods and substantial stakeholder input to explain quantitative analyses, test generalisability and causal inferences and interpret findings in the context of local policy, practice and circumstance.

Quantitative data sources

Health, education and social care data (ECHILD) linked to health visiting data (CSDS)
Our study will exploit national, individual-level health visiting activity data from the CSDS linked with data from children’s social care, hospitals and schools, captured within a new longitudinal dataset known as ECHILD (17). CSDS includes information on individual health visiting contacts, including

For peer review only - http://bmjopen.bmj.com/site/about/guidelines.xhtml

whether the appointment was 'attended' or 'scheduled but not attended', and whether it was a correspondence or face-to-face contact (18). ECHILD links data from hospital admissions, outpatient attendances, A&E and mortality (Hospital Episode Statistics [HES]), education (National Pupil Database) and children's social care services (Child in Need and Looked after Children) for all children in England born from 1995 (17). We will use ECHILD to create longitudinal trajectories for mother and child, capturing information on maternal education, maternal history of contact with children's social care, maternal age at first birth, and number of siblings. Using these data, we will analyse health visiting activity, health and social care utilisation, and outcomes for all children in England aged under five and their mothers, from 2018/19 onwards.

Health visiting taxonomy

As part of an existing study, we are developing a taxonomy of health visiting in all local authorities in England (19). The taxonomy will provide rich descriptions and explanations of 3-5 commonly used models of health visiting, for example: Model 1 – low universal coverage but highly targeted health visiting, conducted mostly by qualified health visitors; Model 2 – high universal coverage but few additional contacts, conducted mostly by band 5 nurses. This classification will be used as an exposure to estimate the association between local models of health visiting and area-level child health and development outcomes.

Aggregate local authority data

We will use Office for Health Improvement & Disparities Fingertips and Interim Reporting Statistics, and The Children's Commissioner's Vulnerability Profiles for aggregate area-level outcomes, where individual-level outcomes are not available (for example, on breastfeeding or vaccination coverage).

Analysis

Scoping

We will conduct a rapid review of literature on health visiting policy, practice and impact. We will gather additional insights through roundtables with policymakers, interviews with practice and commissioning colleagues and workshops with parents. The key aims of these meetings are to a) identify 'priority issues' for different stakeholder groups, which will inform our research questions, analysis and outputs; and b) contribute to our theory of change underpinning the evaluation. The scoping review will ensure that our findings address the needs of policymakers and professionals who commission, manage or deliver health visiting, and reflect what is important to families.

Variation in health visiting contacts

We will use CSDS-ECHILD to describe the number, type (face-to-face, telephone; mandated or additional; home or clinic) and timing of health visiting contacts, and variation by local-area characteristics (e.g. deprivation, rurality) and child/maternal characteristics (e.g. birth order, singleton/multiple birth, number of siblings, maternal education, history of maternal adversity). We will focus our analysis on attended contacts, where the family sees a member of the health visiting team face-to-face. We will also examine 'scheduled but not attended' contacts to determine the extent to which differences in the number of attended contacts between different families or different local areas are due to 'missed' appointments or differences in administrative processes.

We will limit analyses to local authorities for which sufficiently complete health visiting activity data is captured in CSDS, using methods developed in a previous study (7). We will focus on health visiting services as delivered in financial years 2018/19 and 2019/20, as robust data on contacts during the COVID-19 pandemic is not available.

For peer review only - http://bmjopen.bmj.com/site/about/guidelines.xhtml

Impact of health visiting contacts
The impact of health visiting contacts will be assessed for children aged 0-5 years and their mothers at both individual and area level. The exposure for individual-level analysis will be number, type and timing of health visiting contacts. At area level, the exposure will be provided by the local authority taxonomy of health visiting described above.

Outcomes will be defined based on the high impact areas of the HCP (Figure 1):

High impact areas 1&2: Transition to parenthood, maternal and family mental health
Unplanned hospital admissions for mental health, substance misuse or violence; maternal death by suicide; 6-8 week postpartum GP check; timing of maternal mental health diagnosis and prescriptions; prevalence of children in households with severe mental health, domestic violence, or substance addiction

High impact area 3: Supporting breastfeeding
Breastfeeding (6-8 weeks)

High impact area 4: Supporting healthy weight, healthy nutrition
Prevalence of overweight/obesity (reception); decayed, missing or filled teeth (age 5)

High impact area 5: Improving health literacy, reducing accidents and minor illnesses
Infant/child mortality; unplanned admissions for injury/ingestion; unplanned admissions for chronic conditions (asthma, diabetes, epilepsy) and gastroenteritis/lower respiratory tract infections; A&E attendance; Did Not Attend outpatient appointment for chronic conditions; regular GP attendance for children with chronic conditions, including with nurse practitioners; vaccination coverage for MMR and DTaP/IPV/Hib/HepB

High impact area 6: Ready to learn and narrowing the word gap
"Good" level of development and expected communication skills at 2½ year review; "Good" level of development at end of reception

A key challenge for analysing the association between number of health visiting contacts and maternal/child outcomes is that families with higher need may be more likely to receive more intensive health visiting support. A simple comparison of outcomes for families receiving 'high' versus 'low' numbers of health visiting contacts could therefore produce associations between high levels of health visiting and poor outcomes (reverse causality).

To address this problem, we will use propensity score matching to minimise confounding due to differences between groups at baseline, by matching children with similar underlying need (according to maternal and child characteristics) but different numbers of health visiting contacts. This allows us to adjust for the *propensity* of families to receive different numbers or types of health visiting contacts.

One limitation of propensity scores is that we can only match families on known characteristics. In this study, we will have access to a large number of variables: child characteristics at birth (including gestational age, birthweight, ethnicity, area-level deprivation, number of siblings) and maternal characteristics before birth (including age at first birth, history of mental health-related hospital admissions, GCSE-level qualifications, mother's contact with social care services in childhood). However, there may be some remaining confounding due to unmeasured factors such as income and parenting style.

For peer review only

The individual-level analysis will use multi-level regression models (logistic, Poisson or linear) to evaluate the relationship between number and type of health visitor contacts and outcomes in the propensity-matched cohort, accounting for clustering within local authorities. Our primary analysis will be restricted to the earliest birth for each woman from 2018/19 onwards to avoid statistical problems of clustering of children within mothers. To determine whether the effect of health visiting contacts is different for different groups, we will stratify by potential moderators at individual level (e.g. birth order, singleton/multiple birth, ethnicity) and area level (e.g. FNP, Family Hubs, deprivation, ethnic diversity).

In the area-level analysis we will use the taxonomy described above to assign each local authority to a model of health visiting based on health visiting data from 2018/19 and 2019/20. We will create annual aggregated measures of outcomes for each local authority, for years 2018/19-2021/22. We will use linear mixed-effects models to analyse the relationship between health visiting model and outcomes, adjusting for area-level characteristics. This will allow us, for example, to evaluate the effect of model A versus model B of health visiting for children born in 2019 on the proportion of children with a good level of development at the 2-2½ year review in 2021, in local authorities with similar levels of deprivation.

Return on investment analysis

The economic analysis will adhere to the National Institute for Health and Care Excellence (NICE) methods and guidance for the evaluation of public health interventions (20). It will compare total costs to total benefits of different health visiting service delivery models within the local authority taxonomy described above.

The costs of health visiting services will be estimated using CSDS-ECHILD individual-level linked data on health visiting contacts (including duration), combined with unit costs. Unit costs will be obtained using data collected as part of an existing study (19), supplemented with costing information collected from the case study sites (see below). Where not available locally, unit costs for health visiting contacts will be obtained from NHS reference costs (21).

The main return on investment analysis will consider benefits in relation to outcomes for children aged 0-5 years. Outcomes related to contacts with the health system (e.g. A&E attendance) or health outcomes with known long-term financial implications (e.g. obesity) will be valued in monetary terms, using existing local data, information from the case study sites or national sources such as the Personal Social Services Research Unit costs of health and social care (22). To account for potential spillover effects, a secondary analysis will incorporate family and maternal health outcomes such as unplanned maternal hospital admissions for mental health.

We will use the results of these analyses and evidence from the literature to project longer-term benefits in relation to children's obesity, oral health, school exclusions and contact with family or social care services. All outcomes included in the economic analysis are detailed in Figure 1.

Case studies

Case studies will be conducted in up to four local authorities. Using Yin's (23) multiple case study design we will analyse the process of health visiting service delivery in the selected locations, identifying mechanisms of change and determining best local practices that can be applied at a national level. Case study methodology emphasises the importance of context, including geography, demographic profile, local government, health services delivery and individual-level processes such as how health visitors make decisions on type or frequency of contact. This contextual understanding will help to explain why health visiting is delivered variably across England and why there may be different impacts for different groups of families and/or different local areas. Although

For peer review only

COVID-19 is not a direct focus of the study, the qualitative analysis in these case studies will explore the impact of the pandemic on health visiting service delivery and contact types.

Sites will be selected to explain emerging findings from the impact analysis, while seeking maximum variation on population parameters including geography, demographic profile and local government. Data collection will include documentary analysis of local policy and protocols, in-depth interviews with health visiting practitioners, managers and commissioners, and focus groups or interviews with parents.

Interview topic guides will be developed using outputs of the previous stages of the analysis and our discussions with lay stakeholders. We will ask health visitors how they identify families at differing levels of need, and how decisions are made regarding the number, type, and timing of health visiting contacts, by asking about specific families on their case-load (24, 25). We will ask managers and commissioners how health visiting services are organised and delivered locally, and why this is the case. We will ask parents about their experiences and perceptions of health visiting and other relevant services, with prompts on 'priority issues' and important aspects of the theory of change identified in the scoping component. We will work with local stakeholders to gather data on the resources used to provide the health visiting service, which will inform the return on investment analysis.

Integration

This complex, interdisciplinary study uses multiple methods to achieve its objectives (Figure 2). It is widely recognised that integrating qualitative and quantitative data within a single study can bring unique insights, allowing more nuanced and deeper analysis of complex phenomena (such as health visiting) than studies using a single method (26, 27). Achieving these benefits of a mixed methods study relies on successfully integrating the data at the level of study design, analysis, interpretation and presentation (28). At study design level, we will adopt an 'advanced multistage mixed methods framework' (29) combining three core approaches:

Exploratory sequential: The qualitative data collected through the scoping component will establish priority issues and generate hypotheses to pursue in the quantitative component and in the case studies.

Explanatory sequential: The qualitative interview data collected through case studies will help explain any variation in health visiting and the differential effects of health visiting that we find in the quantitative analyses

Convergent: The area-level impact component will overlap with the case studies, and emerging findings will be tested for confirmation, expansion or divergence.

At analysis and interpretation stages, we will take a 'constant comparative approach'. This is a hallmark of Grounded Theory (a qualitative research method) but can be employed across data types and paradigms (30). We will hold regular 'integration meetings' where team members come together to compare challenges, insights and emerging findings across study components. All research outputs will draw on multiple components of the study, with priority issues and theory generated from the case studies playing a key role in integrating the findings throughout.

Patient and Public Involvement

We have taken into account the views and experiences of parents when planning this study, via four PPI sessions. Participants were aware that experiences of health visiting differed significantly

For peer review only - http://bmjopen.bmj.com/site/about/guidelines.xhtml

between families and local areas and there was a strong message that reasons for variation should be understood. Parents highlighted how important it was to see the same health visitor (continuity of care) and to build a good relationship with 'their' health visitor. These concerns have informed our case study design, where we will conduct ‘deep dives’ into selected local areas to understand what drives variation, and the importance of the parent-professional relationship as a mechanism of effect.

For this study we will establish three PPI groups: a virtual group of eight parents, and a group of mothers and a group of fathers from Barking and Dagenham in northeast London, with up to six participants in each. We will consult these groups at two key times, initially during the scoping review and towards the end of the study to help with interpretation and communication of findings.

ETHICS AND DISSEMINATION

Ethics

This study has been approved by University College London Research Ethics Committee (ref 20561/002) and the National Research Ethics Service (21/SW/0159). Access to ECHILD, HES and CSDS has been approved by NHS England (NIC-393510 and NIC-381972). The case study element involves NHS access and separate approval is being sought from the Health Research Authority.

Dissemination

The findings of this study will be of benefit to national policy makers, local directors of public health, commissioners and managers of health visiting services, health visitors and parents. Our research will inform national guidance on the health visiting component of the HCP, contributing to the modernisation of the HCP by the Department of Health and Social Care and the Office for Health Improvement and Disparities. Our findings will be used by local leaders as they develop and improve their Start for Life offer and their local HCP. Evidence about the impact, costs and benefits of health visiting services will inform submissions to the 2024 Spending Review.

We will produce policy briefings aimed at health visiting decision-makers and practitioners, and lay summaries, which can be used to raise awareness among parents of the purpose of health visiting and what they can expect from their contacts with health visitors. For researchers, we aim to publish our findings in peer-reviewed journal articles and present at key conferences.

For peer review only

FOOTNOTES

Contributors: KH and JW are the coprincipal investigators of the study and designed the study with input from FC, AC, SK, RC, JA, E-CS and HB. FC and CB produced the first draft of this manuscript, with input from KH, AC, SK, RC, JA, E-CS, HB and JW. All authors edited the manuscript. All authors contributed to and are responsible for the final design of the study. All authors read and approved the final manuscript.

Funding: This work is supported by the National Institute for Health Research (NIHR) Health Services Policy Research Programme (grant number NIHR203450). This research was supported in part by the NIHR Great Ormond Street Hospital Biomedical Research Centre.

Disclaimer: The views expressed are those of the author(s) and not necessarily those of the NIHR or the Department of Health and Social Care.

Competing interests: None declared

Provenance and peer review: Not commissioned; peer reviewed for ethical and funding approval prior to submission.

For peer review only

REFERENCES

1. Public Health England. Health visiting and school nursing service delivery model - Guidance 2021 [Available from: https://www.gov.uk/government/publications/commissioning-of-public-health-services-for-children/health-visiting-and-school-nursing-service-delivery-model.
2. Woodman J, Simon A, Hauari H, Gilbert R. A scoping review of 'think-family' approaches in healthcare settings. Journal of public health (Oxford, England). 2020;42(1):21-37.
3. Public Health England. Rapid review to update evidence for the Healthy Child Programme 0-5. London; 2015.
4. Asmussen K, Feinstein L, Martin J, Chowdry H. Foundations for life: What works to support parent child interaction in the early years. London: EIF; 2016.
5. Public Health England. Health visitor service delivery metrics: 2018/19 annual data statistical commentary. 2019.
6. Public Health England. Characteristics of children receiving universal health visitor reviews 2021 [Available from: https://www.gov.uk/government/publications/characteristics-of-children-receiving-universal-health-visitor-reviews.
7. Fraser C, Harron K, Barlow J, Bennett S, Woods G, Shand J, et al. How can we use the community services dataset (CSDS) for research into health visiting? 2020 [Available from: https://www.ucl.ac.uk/children-policy-research/sites/children-policy-research/files/using_csds_for_research_report_08.10.20.pdf.
8. Public Health England. Contacts other than those mandated and referrals by health visitors - Experimental analysis of the Community Services Dataset. 2021.
9. Black M, Barnes A, Baxter S, Beynon C, Clowes M, Dallat M, et al. Learning across the UK: a review of public health systems and policy approaches to early child development since political devolution. Journal of Public Health. 2019;42(2):224-38.
10. Institute of Health Visiting. Health Visiting in England: A Vision for the Future 2019 [Available from: https://ihv.org.uk/wp-content/uploads/2019/11/7.11.19-Health-Visiting-in-England-Vision-FINAL-VERSION.pdf.
11. Goldfeld S, Bryson H, Mensah F, Gold L, Orsini F, Perlen S, et al. Nurse Home Visiting and Maternal Mental Health: 3-Year Follow-Up of a Randomized Trial. Pediatrics. 2021;147(2):e2020025361.
12. Asmussen K, Brims L. What works to enhance the effectiveness of the Healthy Child Programme: An Evidence Update. London; 2018.
13. Kemp L, Harris E, McMahon C, Matthey S, Vimpani G, Anderson T, et al. Child and family outcomes of a long-term nurse home visitation programme: a randomised controlled trial. Archives of Disease in Childhood. 2011;96(6):533-40.
14. Cavallaro F, Gilbert R, van der Meulen J, Kendall S, Kennedy E, Harron K. Evaluation of Intensive Home Visiting for Adolescent Mothers in the Family Nurse Partnership: A Population Based-Data Linkage Cohort Study in England. Available from: https://ssrn.com/abstract=4337372.
15. Conti G, Dow A. Using FOI data to assess the state of Health Visiting Services in England before and during COVID-19 London: UCL Department of Economics; 2021 [Available from: https://discovery.ucl.ac.uk/id/eprint/10132710/1/Conti_Dow_COVID-19_Health_Visiting_FOI1_POSTING_Aug.2021.pdf.
16. Tariq S, Woodman J. Using mixed methods in health research. JRSM Short Reports. 2013;4(6):2042533313479197.
17. Mc Grath-Lone L, Blackburn R, Gilbert R. The Education and Child Health Insights from Linked Data (ECHILD) Database: An Introductory Guide for Researchers 2021 [Available from: https://www.ucl.ac.uk/child-health/sites/child-health/files/echild_user_guide_v1.1.0.pdf.

For peer review only - http://bmjopen.bmj.com/site/about/guidelines.xhtml

18. Fraser C, Harron K, Barlow J, Bennett S, Woods G, Shand J, et al. Variation in health visiting contacts for children in England: cross-sectional analysis of the 2-2(1/2) year review using administrative data (Community Services Dataset, CSDS). BMJ Open. 2022;12(2):e053884.
19. Woodman J, Mc Grath-Lone L, Clery A, Weatherly H, Jankovic D, Appleton JV, et al. Study protocol: a mixed-methods study to evaluate which health visiting models in England are most promising for mitigating the harms of adverse childhood experiences. BMJ Open. 2022;12:e066880.
20. National Institute for Health and Care Excellence. Methods for the Development of NICE Public Health Guidance London: National Institute for Health and Care Excellence (NICE). Process and Methods Guides No. 4; 2012 [Available from: https://www.nice.org.uk/process/pmg4/chapter/incorporating-health-economics
21. Department of Health and Social Care. National Schedule of Reference Costs Year 2018–2019 London: Department of Health and Social Care; 2018 [Available from: https://www.england.nhs.uk/publication/2018-19-national-cost-collection-data-publication/
22. Curtis L, Burns A. Unit Costs of Health and Social Care 2018: Personal Social Services and Research Unit, University of Kent, Canterbury; 2018.
23. Yin R. Case study research and applications: Design and methods. Sixth edition: Sage publications; 2018.
24. Public Health England. Best start in life and beyond: improving public health outcomes for children, young people and families: guidance to support the commissioning of the Health Child Programme 0-19: Health Visiting and school nursing services. Commissioning guide 1: background information on comissioning and service model. London; 2018.
25. Department for Health. Healthy Child Programme. Pregnancy and the First Five Years. London; 2009.
26. O'Cathain A, Murphy E, Nicholl J. Three techniques for integrating data in mixed methods studies. BMJ. 2010;341:c4587.
27. Castro FG, Kellison JG, Boyd SJ, Kopak A. A Methodology for Conducting Integrative Mixed Methods Research and Data Analyses. J Mix Methods Res. 2010;4(4):342-60.
28. Guetterman TC, Fetters MD, Creswell JW. Integrating Quantitative and Qualitative Results in Health Science Mixed Methods Research Through Joint Displays. Ann Fam Med. 2015;13(6):554-61.
29. Fetters MD, Curry LA, Creswell JW. Achieving integration in mixed methods designs-principles and practices. Health services research. 2013;48(6 Pt 2):2134-56.
30. Mathison Se. Constant Comparative Method. Encyclopedia of Evaluation. Thousand Oaks, California2005.

Figure 1 Overview of study outcomes

Figure 2 Integration of study components

For peer review only

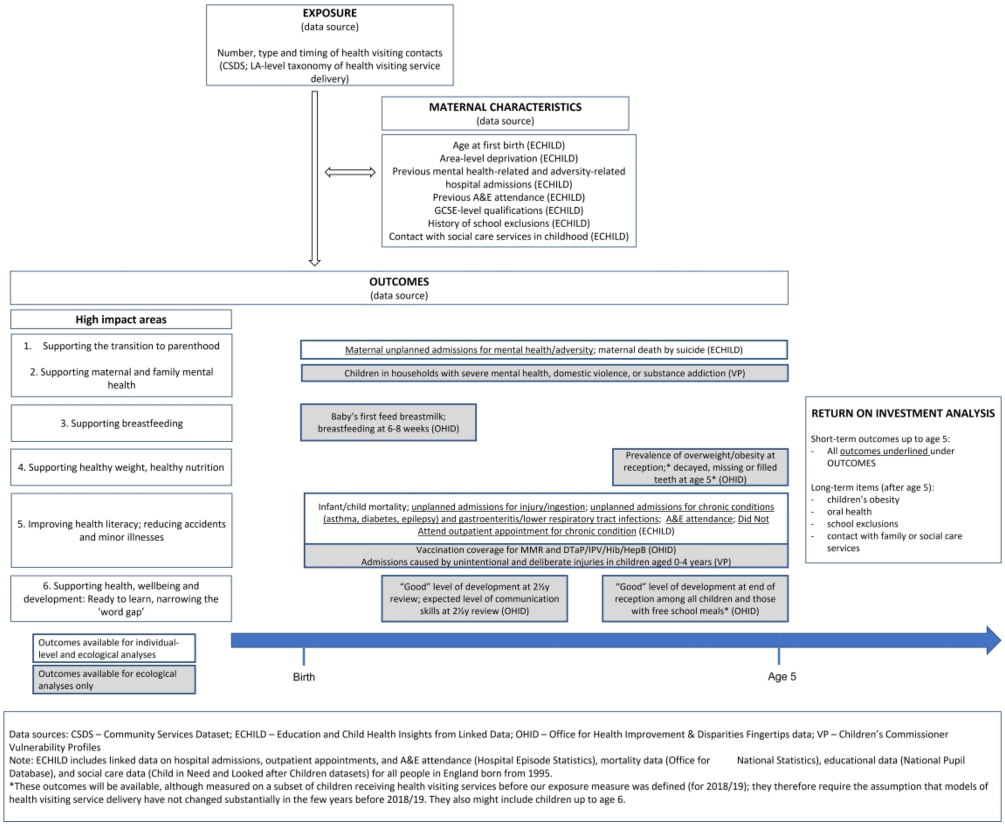

Figure 1. Overview of study outcomes

488x400mm (330 x 330 DPI)

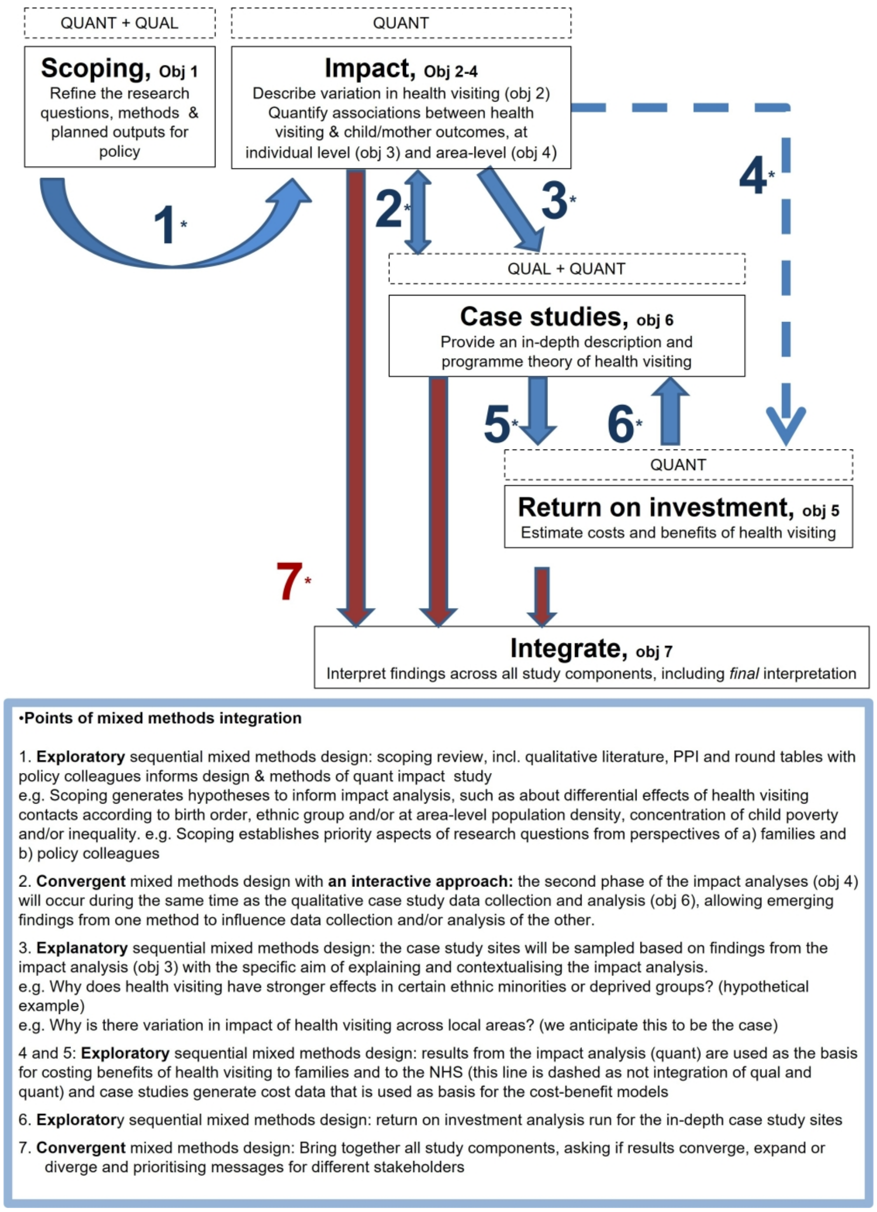

Figure 2. Integration of study components

210x289mm (330 x 330 DPI)
