# Supplementary material for: Study protocol: evaluation of the 0–5 public health investment in England – a mixed-methods study integrating analyses of national linked administrative data with in-depth case studies
Source: BMJ Open. 2023 Apr 5;13(4):e073313. doi: 10.1136/bmjopen-2023-073313 (PMC10083857; doi:10.1136/bmjopen-2023-073313)
Supplement: Reviewer comments [file bmjopen-2023-073313.reviewer_comments.pdf]

PEER REVIEW HISTORY

BMJ Open publishes all reviews undertaken for accepted manuscripts. Reviewers are asked to complete a checklist review form (**http://bmjopen.bmj.com/site/about/resources/checklist.pdf**) and are provided with free text boxes to elaborate on their assessment. These free text comments are reproduced below.

ARTICLE DETAILS

**TITLE (PROVISIONAL)**	Study protocol: evaluation of the 0-5 Public Health Investment in England: a mixed methods study integrating analyses of national linked administrative data with in-depth case studies
**AUTHORS**	Harron, Katie; Cavallaro, Francesca; Bunting, Catherine; Clery, Amanda; Kendall, Sally; Cassidy, Rebecca; Atkins, Julie; Saloniki, Eirini-Christina; Bedford, Helen; Woodman, Jenny

This article was not externally reviewed at BMJ Open. Protocols that have been independently assessed prior to submission to BMJ Open are usually fast-tracked to publication on the grounds that further substantial changes will not be possible. This independent assessment will usually be external, independent review for both a substantial grant award from a non-commercial or government funder and ethics approval.
